# Milk-flow data collected routinely in an automatic milking system: an alternative to milking-time testing in the management of teat-end condition?

**DOI:** 10.1186/s13028-018-0356-x

**Published:** 2018-01-11

**Authors:** Håvard Nørstebø, Amira Rachah, Gunnar Dalen, Odd Rønningen, Anne Cathrine Whist, Olav Reksen

**Affiliations:** 1 Department of Production Animal Clinical Sciences, Faculty of Veterinary Medicine, Norwegian University of Life Sciences, PO Box 8146 Dep, 0033 Oslo, Norway; 2 TINE SA, Langbakken 20, 1430 Ås, Norway

**Keywords:** Automatic milking, Milk flow, Teat-end callosity

## Abstract

**Background:**

Having a poor teat-end condition is associated with increased mastitis risk, hence avoiding milking machine settings that have a negative effect on teat-end condition is important for successful dairy production. Milking-time testing (MTT) can be used in the evaluation of vacuum conditions during milking, but the method is less suited for herds using automatic milking systems (AMS) and relationships with teat end condition is poorly described. This study aimed to increase knowledge on interpretation of MTT in AMS and to assess whether milk-flow data obtained routinely by an AMS can be useful for the management of teat-end health. A cross-sectional study, including 251 teats of 79 Norwegian Red cows milked by AMS was performed in the research herd of the Norwegian University of Life Sciences. The following MTT variables were obtained at teat level: Average vacuum level in the short milk tube during main milking (MTVAC), average vacuum in the mouthpiece chamber during main milking and overmilking, teat compression intensity (COMPR) and overmilking time. Average and peak milk flow rates were obtained at quarter level from the AMS software. Teat-end callosity thickness and roughness was registered, and teat dimensions; length, and width at apex and base, were measured. Interrelationships among variables obtained by MTT, quarter milk flow variables, and teat dimensions were described. Associations between these variables and teat-end callosity thickness and roughness, were investigated.

**Results:**

Principal component analysis showed clusters of strongly related variables. There was a strong negative relationship between MTVAC and average milk flow rate. The variables MTVAC, COMPR and average and peak milk flow rate were associated with both thickness and roughness of the callosity ring.

**Conclusions:**

Quarter milk flow rate obtained directly from the AMS software was useful in assessing associations between milking machine function and teat-end condition; low average milk flow rates were associated with a higher likelihood of the teat having a thickened or roughened teat-end callosity ring. Since information on milk flow rate is readily available from the herd management system, this information might be used when evaluating causes for impaired teat-end condition in AMS.

## Background

Changes in condition of the teat-end of dairy cattle, as evaluated by thickness and roughness of the callosity ring, have been associated with increased mastitis risk in previous studies from conventional milking systems (CMS) [1, 2]. Factors related to milking machine function, environment such as housing and climate, and general management have been identified as risk factors for alterations of the integrity of teat tissue [3]. One major advantage of automatic milking systems (AMS) over CMS is a reduction of overmilking by individual attachment and detachment of the four teat cups [4, 5]. However, thickening of the skin surrounding the teat orifice, teat-end callosity, is still found also in AMS herds, necessitating further studies on how to manage the problem. AMS was first introduced in Norwegian dairy herds in 2000 [6], and in 2017 more than 30% of Norwegian dairy farms had adopted this technology [7]. AMS continuously records large amounts of data from the milking process. Using such data in decision-support systems enabling the farmer to improve herd health has been subject to extensive research the last decade [8]. This approach has motivated us to explore whether improved utilization of data from the AMS might be useful when investigating causes for impaired teat end condition in AMS herds.

Milking-time tests (MTT) are used under field conditions to assess the potential negative effects of the milking equipment on the teat. MTT is defined as a “test made on a milking machine during milking of live animals” [9]. However, documentation on how MTT variables relate to each other, and to teat-end condition, is limited in AMS herds because most research on the topic has been performed in CMS [10]. Performing MTT in AMS herds is also time demanding because only one cow is milked at a time, making the method less practical.

Several parameters have been established to evaluate the forces applied on the teat by the collapsing liner [11–13]. Spohr and Uhlenbruck [12] described a variable called “drucksumme” (German; “sum of pressure”), estimating the forces acting on the teat-end during the closed phase of the pulsation cycle by using milking-time vacuum recordings from the short milk tube and pulsation tube, and found a strong positive correlation between “drucksumme” and percentage of cows with high degree of teat-end callosity [12]. However, an association between “drucksumme” and teat-end condition on quarter level has not yet been reported.

The vacuum level in the short milk tube, and hence at the teat-end, is one of many parameters used in a standard MTT. This vacuum level is influenced mainly by the system vacuum level and the milk transport through the short and long milk tube [14]. Quarter milk flow rate is related to system vacuum level [15, 16], teat anatomy [17] and pulsation settings [18]. It seems reasonable to assume that for cows milked by the same milking system (i.e. same system vacuum, pulsation settings and liner), individual cow- or teat factors are responsible for variation in vacuum level in the short milk tube, and hence the forces applied on the teat.

The vacuum level in the mouthpiece chamber (MPC) is another parameter often obtained by MTT. MPC vacuum is a proposed measurement for how well the teat fits the liner [19]. A recent study showed that a high MPC vacuum was associated with congestion at the teat-end [20]. However, a possible association between MPC vacuum and long-term changes in teat-end condition, such as teat-end callosity, has not been evaluated so far.

The overall aim of this study was to assess whether data obtained routinely by the AMS can be used as a proxy for MTT variables in the management of teat-end condition in AMS herds. Our first objective was to describe interrelationships between variables from MTT, the AMS and teat dimension measurements. Secondly, we wanted to assess relationships between these variables and teat-end callosity thickness and roughness, and finally to compare the fit of these models to conclude on the overall aim.

## Methods

### Herd description and milking machine settings

Our study was performed in the research herd at the Norwegian University of Life Sciences. The herd consisted of 91 Norwegian Red cows divided in two groups, each milked by one AMS (DeLaval VMS, Tumba, Sweden) with identical settings. These 91 cows formed the source population. The two groups were situated in immediate proximity and had identical housing conditions. The teatcups were equipped with DeLaval 20 M VMS liner (Product No. 92725901). The system vacuum was set to 45 kPa, the pulsator ratio was 65% and the pulsation rate was 60 cycles/min. Quarter take-off limit (switch-level) was set to 0.24 kg/min. The system was set with a low-vacuum period of 6 s and a delay from initiation of detachment to detachment of 4 s. The herd, including housing conditions, management and equipment, is comparable to commercial AMS farms in Norway. Data on parity and days in milk (DIM) in the study herd were obtained from the Norwegian Dairy Herd Recording System [21]. Except from a period of 3–5 days immediately after calving, the included cows had not been milked by CMS in the current lactation. Cows in second or later lactations had been milked by CMS in previous lactations.

### Teat-end scoring and teat dimensions

Teats on all 91 lactating cows in the herd were evaluated using a scoring system where the thickness and roughness of the callous ring of the teat orifice was categorized into one of eight classes [22]. Teat length (Length) and width 0.5 cm from apex (Apex) and base (Base) was measured by placing the teat between a white background and a transparent 0.5 cm grid (DeLaval, Tumba, Sweden). In addition, the shape of the teat-end was classified in the following groups; pointed, round, flat or inverted, and the teat position was registered. All registrations were performed once per cow during a 2-day period. The udder and teats were cleaned and stripped prior to the evaluation. The same person performed all scoring and registrations. The time from last milking to teat evaluation was not standardized.

Two outcome variables for logistic regression models were established by transforming the results from the teat-end scoring. The variable THICKNESS was given the value 0 if the thickness of the callosity ring was thin or not visible, and the value 1 if it was classified as medium, thick or extreme according to the scoring system by Neijenhuis et al. [22]. The variable ROUGHNESS was given the value 0 if a smooth callosity ring was registered, and the value 1 if a rough callosity ring was registered. This approach was chosen because previous research has shown that severe degrees of teat-end callosity is significantly related to mastitis risk [2, 23].

### Vacuum recordings

VaDia vacuum recorders (BioControl, Rakkestad, Norway) were used to record vacuum at a rate of 200 Hz in the short milk tube, pulsation tube, and MPC in the four individual teatcups during milking in both milking stations. The data were collected during three herd visits, between 1 and 4 weeks after the initial teat-end scoring was performed. We used data from a convenience sample of cows that entered the milking stations voluntarily without interference from the herdsmen. For cases of duplicate vacuum registrations of the same quarter, the recording taken closest to the day of teat-end scoring was used. Cows not entering the milking stations during our visits were excluded from the study. Teats that had been dried-off prior to our observational period and teats where MTT variables could not be calculated due to missing vacuum recordings were also excluded.

### Calculation of MTT variables per quarter

In accordance with common procedures for MTT under field conditions, the different periods of the milking were found by evaluating the vacuum recordings [24]. For each milking two main periods were identified, based on the vacuum registrations from the short milk tube and the MPC: (a) the main milking period and (b) the overmilking period. The main milking period was characterized by high milk flow and stable vacuum conditions in the short milk tube. The start of the main milking period was identified by monitoring the average short milk tube vacuum in 10 s periods, until the decline from one period to the next was less than 0.3 kPa. The end of the main milking period, which coincides with the start of the overmilking period, was defined as the point where a marked change in MPC vacuum became apparent as described by Borkhus and Rønningen [19]. Automatic detection of these MPC vacuum changes was set at the point where the MPC vacuum increased at least 30% plus 2 kPa above a weighted running average. The result of the automated procedure was controlled manually and if necessary adjusted taking changes in short milk tube vacuum into account as described previously [19]. The end of the overmilking period was set to the initiation of detachment, i.e. the point where the short milk tube vacuum started to fall markedly (≥ 5 kPa) shortly before the end of milking.

The average vacuum in the short milk tube during the main milking period (MTVAC) was calculated as the mean of all vacuum recordings from the short milk tube within the main milking period. The average vacuum levels in the MPC during the main (MPCVAC) and the overmilking periods (MPCOM) were calculated accordingly.

To estimate the forces applied on the teat-end by the liner during the closed-phase of the pulsation cycle, the variable teat compression intensity (COMPR) was calculated for each teat. This variable is comparable with “drucksumme”, as described by Spohr and Uhlenbruck [12]. Vacuum records from the pulsation tube and short milk tube of 10 consecutive pulsation cycles from 60 to 70 s into the main milking period were used for the calculation of COMPR. Differential pressure across the liner wall, i.e. the difference between the short milk tube vacuum and the pulsation tube vacuum, was calculated throughout the 10 pulsation cycles. Touchpoint pressure difference (TPPD) is the pressure difference across the liner wall when two sides of the liner achieves or loses contact during closing and opening respectively [9]. TPPD is traditionally measured without a teat in the teatcup [25]. In this study, however, approximated values for TPPD was derived from the pulsation tube vacuum curves at the points of fastest liner wall movement during opening and closing, respectively [26]. The average of the approximated TPPD at opening and closing was used in further calculations. The closed-phase of the pulsation cycles were defined as the period when the differential pressure across the liner wall was higher than the approximated TPPD. For each of the 10 pulsation cycles, an integral of the differential pressure across the liner wall minus approximated TPPD as a function of time over the liner closed period was calculated. The average of the integrals found in the 10 pulsation cycles represented teat compression intensity, COMPR (kPa s).

Quarter average milk flow rate (AVGFLOW) and quarter peak milk flow rate (PEAKFLOW), for the same milkings in which the vacuum measurements were performed, were obtained from the AMS software (DeLaval, Tumba, Sweden). The milking stations were equipped with ICAR-approved milk meters using near-infrared technology providing in-line data on milk flow and milk yield used in the calculation of these variables.

### Statistical analyses

Principal component analysis (PCA) is a multivariate technique that can be used to explore multi-dimensionality of data and to reduce a large set of variables to a small number of latent variables, principal components, which nevertheless retain most of the information in the dataset. PCA is also useful for the analysis of intercorrelation of variables. We applied PCA to study the relationships between the variables obtained from the different registrations, and as suggested by Dohoo et al. [27], we used PCA as a complementary technique in the subsequent model-building process. We used the 2-dimensional scatter plot of loadings for two specified components from PCA, which is most useful for interpreting principal component 1 versus principal component 2, as they contain the most important information in the data. In our application of PCA, we focused on the geometric interpretation of the relationships between variables, plotted as points in the component space using their loadings as coordinates on the “circle of correlations”. In addition to PCA, we also used a linear regression model to describe the mathematical relationship between MTVAC and AVGFLOW.

The following variables were evaluated as potential explanatory variables in logistic regression models describing the likelihood of a teat-end having a rough or thickened callosity ring, respectively: DIM, Parity, MTVAC, COMPR, AVGFLOW, PEAKFLOW, MPCVAC, MPCOM, Length, Apex, Base, teat position and overmilking time. Parity was categorized as; first lactation, second lactation, and greater than second lactation.

Linearity between the outcome variables and the explanatory variables was assessed by inspecting lowess smoothing curves obtained with a logit transformation of the outcome variables; THICKNESS and ROUGHNESS (Stata SE/14, Stata Corp., College Station, TX, USA).

To establish logistic regression models for teat-end callosity roughness and thickness, we initially tested each of the explanatory variables in univariable logistic regression models to detect associations with the two outcome variables, THICKNESS and ROUGHNESS. Variables with a P-value less than 0.2 were further evaluated for inclusion in multivariable models. In order to avoid including highly correlated variables in the same model, variables identified by PCA as belonging either to the same cluster or in clusters aligned on the opposite side of the circle of correlation, were evaluated in separate models. We repeated a backwards selection procedure to build multiple multivariable models for each outcome, and variables with P-value ≥ 0.05 were excluded from the final multivariable models. Because teat-end callosity thickness and roughness has been shown to vary between parities and lactation stages, parity and DIM were forced into all multivariable models [1].

We expected registrations between teats within the same cow to be correlated, and to account for this, we included a random intercept at cow level in both the univariable and multivariable models. Because the multivariable models were non-nested, i.e. the predictors in one model could not be considered as subsets of the predictors in other models, Bayesian Information Criterion (BIC) was calculated to compare model fit [27].

Data from different sources were assembled in SAS 9.4 (SAS Institute Inc., Cary, NC, USA) to form a final dataset. We used the STATA meqrlogit procedure for the logistic regression analysis and the regress procedure for the linear regression analysis (Stata SE/14, Stata Corp., College Station, TX, USA). The PCA was conducted using the statistical software JMP Pro version 12 (SAS Institute Inc., Cary, NC, USA).

## Results

Out of 91 cows that were assigned teat-end scores, eight cows did not enter the milking stations at any of the visits for MTT. Of the remaining 83 cows, complete vacuum registrations from at least one teat were obtained in 79 cows, while four cows were excluded due to complete or partial loss of vacuum data for all teats. From 79 cows, four teats were excluded because they were dried off, while 61 teats were excluded due to complete or partial loss of vacuum data. This resulted in a study sample including 251 teats in 79 cows.

The parities in the study sample were distributed as follows; 34 cows in first lactation, 13 in second, and 32 in third or higher lactations. Table 1 shows the distribution of teat-end callosity scores in the included teats. The data included 123 front teats and 128 rear teats. Concerning teat-end shape, 145 teat-ends were classified as round, 78 flat, 12 pointed and 16 inverted. The number of roughened teat-ends in the same groups were 54, 15, 9 and 0, respectively. Due to the small number of observations with inverted and pointed teats, the teat-end shape was not included in statistical models. The average DIM at the time of teat-end scoring was 76, ranging from 4 to 167. The average overmilking time was 32 s, and ranged from 16 s to 3 min 17 s. However, 90% of the observations had an overmilking time shorter than 48 s.

**Table 1:** Frequency of teat-end callosity scores [22] in the study herd

Callosity ring thickness	Callosity ring roughness		Total
	Smooth	Rough	
Not visible	53	–	53
Thin	80	37	117
Intermediate	30	31	61
Thick	10	8	18
Extremely thick	–	2	2
Total	173	78	251

### Relationships among milk flow-variables, MTT-variables and teat dimensions

**Fig. 1:**
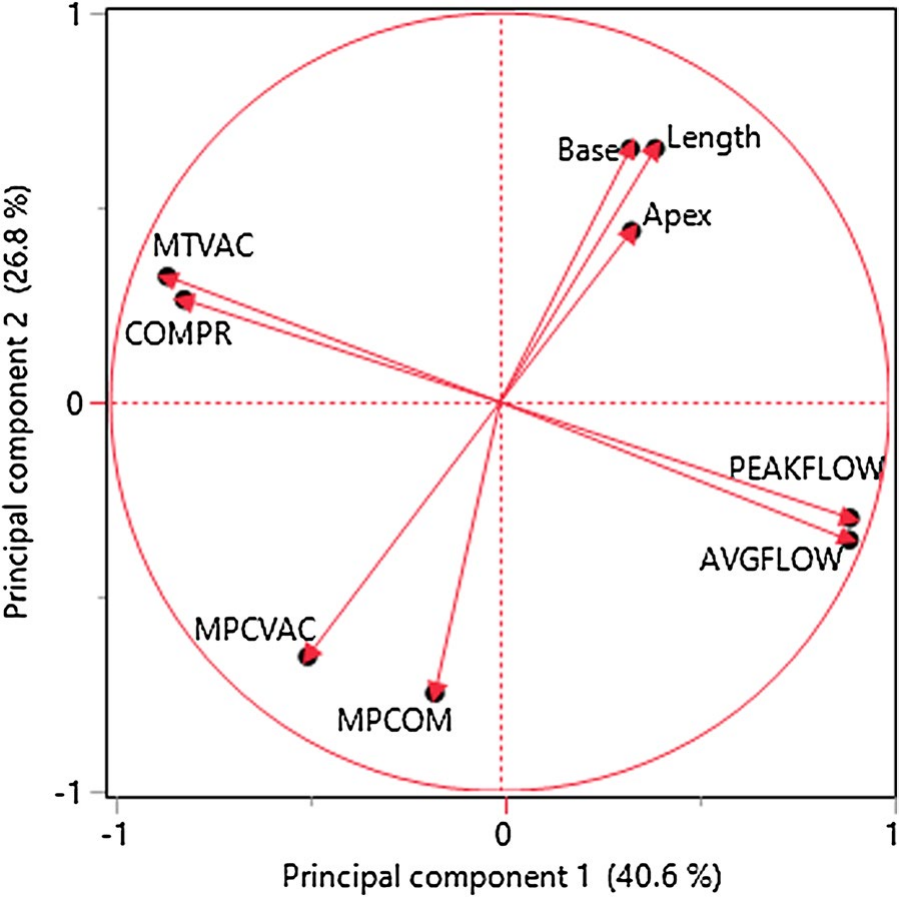
Principle component loading plot. Loading plot, describing the relationship between milking-time test variables and teat characteristics, derived from principal component analysis. From this loading plot, we distinguished 4 clusters of variables: cluster 1, consisting of MPCVAC and MPCOM; cluster 2, consisting of Length, Apex, and Base; cluster 3, including AVGFLOW and PEAKFLOW; cluster 4, consisting of MTVAC and COMPR

Figure 1 shows the plot of the loadings of the explanatory variables MTVAC, COMPR, MPCVAC, MPCOM, PEAKFLOW AVGFLOW, Length, Apex and Base on the components. Each variable is a point whose coordinates are given by the loadings on the principal components. The first and second principal components described 67.4% of the total variation of these explanatory variables. From this loading plot we distinguished 4 clusters of variables: Cluster 1, consisting of MPCVAC and MPCOM; cluster 2, consisting of Length, Apex, and Base; cluster 3, including AVGFLOW and PEAKFLOW; cluster 4, consisting of MTVAC and COMPR. Cluster 1 loaded opposite to cluster 2, showing a negative relationship between teat dimensions and vacuum levels in the MPC. MTVAC and COMPR in cluster 4 were negatively correlated with PEAKFLOW and AVGFLOW of cluster 3.

A linear regression model showed a strong linear relationship between MTVAC and AVGFLOW with a coefficient of determination (R^2^) of 0.71. The relationship was described mathematically by the following equation:

$$MTVAC = 42.9 - 0.38 \times AVGFLOW$$

The R^2^ increased to 0.84 when we omitted the 3 observations with the largest residuals.

### Relationships between teat-end callosity and milk flow- and MTT-variables

In the univariable analysis, the outcome variable ROUGHNESS was significantly associated with AVGFLOW, PEAKFLOW, COMPR and MTVAC (P < 0.05). No significant association was found between ROUGHNESS and teat position. The results of the univariable analyses are presented as odds ratios in Table 2. Cows with teat-ends classified as normal were the designated comparison group and were assigned the odds ratio (OR) value of 1. OR are multiplicative measures of risk that range from 0 to infinity. OR > 1 is predisposing and implies an increased risk. OR < 1 is preventive and implies an inverse association.

**Table 2:** Results from univariable analysis for teat-end callosity roughness and thickness

Variable	ROUGHNESS				THICKNESS			
	Odds ratio	P	95% CI		Odds ratio	P	95% CI	
			Lowe	Upper			Lower	Upper
MTVAC, kPa	2.126	0.001	1.357	3.331	1.890	0.008	1.185	3.016
COMPR, kPa s	1.380	0.014	1.069	1.781	1.443	0.016	1.072	1.942
AVGFLOW, kg/min	0.040	0.001	0.006	0.265	0.049	0.005	0.006	0.406
PEAKFLOW, kg/min	0.082	0.001	0.018	0.382	0.146	0.022	0.028	0.761
MPCVAC, kPa	1.008	0.708	0.967	1.050	0.965	0.132	0.921	1.011
MPCOM, kPa	0.979	0.480	0.922	1.039	0.928	0.040	0.865	0.997
Length, cm	1.137	0.665	0.635	2.034	1.054	0.874	0.549	2.024
Apex, cm	0.241	0.099	0.045	1.305	0.843	0.853	0.138	5.136
Base, cm	0.907	0.865	0.295	2.793	1.252	0.714	0.376	4.170
Overmilking time, min	0.916	0.858	0.352	2.384	0.558	0.402	0.143	2.182

MTVAC, average vacuum level in the short milk tube during the main milking period; COMPR, teat compression intensity; AVGFLOW, quarter average milk flow rate; PEAKFLOW, quarter peak milk flow rate; MPCVAC, average vacuum level in the mouthpiece chamber during the main milking period; MPCOM, average vacuum level in the mouthpiece chamber in the overmilking period. Random intercepts at cow level were included in all analyses to account for within cow dependency of the outcome variables

ROUGHNESS, dichotomized outcome variable where smooth teat-end callosity rings form the comparison group and teat-ends with a roughened callosity ring is considered abnormal

THICKNESS, dichotomized outcome variable where teat-ends having a thin or not visible teat-end callosity ring form the comparison group, and medium, thick or extreme are considered abnormal

The results from the PCA indicated that the variables AVGFLOW, PEAKFLOW, COMPR and MTVAC were strongly related. To avoid collinearity in the multivariable models, separate model building procedures were performed by including one of these variables in addition to remaining variables from the univariable analyses meeting the inclusion criteria.

The results of the multivariable models showed that MTVAC, COMPR, AVGFLOW and PEAKFLOW were all associated with ROUGHNESS (P < 0.01). The model with AVGFLOW as explanatory variable (Table 3) had the lowest BIC (290.59). The models using MTVAC, COMPR and PEAKFLOW had a BIC of 290.70, 297.95 and 291.88, respectively.

**Table 3:** Final multivariable logistic regression models describing the likelihood of a teat having a roughened or thickened teat-end callosity ring, respectively [22]

Outcome and BIC	Variable	Odds ratio	P	95% CI	
				Lower	Upper
ROUGHNESS	DIM	1.016	0.032	1.001	1.030
BIC = 290.59	Parity 1 (reference)	–	–	–	–
	Parity 2	0.291	0.201	0.044	1.928
	Parity ≥ 3	2.943	0.073	0.903	9.593
	AVGFLOW, kg/min	0.020	0.001	0.003	0.160
THICKNESS	DIM	1.024	0.006	1.007	1.041
BIC = 281.38	Parity 1 (reference)	–	–	–	–
	Parity 2	0.386	0.381	0.046	3.249
	Parity ≥ 3	3.969	0.056	0.963	16.362
	AVGFLOW, kg/min	0.019	0.001	0.002	0.181

The models were selected based on having the lowest Bayesian information criterion (BIC) among other models for the same outcome

DIM, days in milk; AVGFLOW, quarter average milk flow rate

ROUGHNESS, dichotomized outcome variable where smooth teat-end callosity rings form the comparison group and teat-ends with a roughened callosity ring is considered abnormal

THICKNESS, dichotomized outcome variable where teat-ends having a thin or not visible teat-end callosity ring form the comparison group, and medium, thick or extreme are considered abnormal

For the outcome variable THICKNESS, the univariable analysis showed a significant association with AVGFLOW, PEAKFLOW, COMPR, MTVAC and MPCOM (P < 0.05). The results are presented in Table 2. A significant association was not found between THICKNESS and teat position.

MTVAC, COMPR, AVGFLOW and PEAKFLOW were significantly associated with THICKNESS in multivariable models (P < 0.01). The model with AVGFLOW as explanatory variable had lowest BIC (281.38) also for this outcome (Table 3). BIC for the models using MTVAC, COMPR and PEAKFLOW were 283.22, 282.86 and 285.33, respectively.

The random intercept term signifying the correlation between teats within the same cow was highly significant (P < 0.001) in all models.

## Discussion

### Relationships among milk flow-variables, MTT-variables and teat dimensions

The clustering of variables identified by the PCA shows that recording and evaluating a smaller number of variables might be sufficient for MTT in AMS herds. Cluster 1 was based on vacuum recordings from the MPC, cluster 2 represented the measured teat dimensions, cluster 3 displayed milk flow recordings, and cluster 4 was based on vacuum recordings from the short milk tube.

We observed a strong negative relationship between MTVAC and AVGFLOW. This is in agreement with previous studies [16, 28]. The relationship between system vacuum, claw vacuum and milk flow has been described in previous experimental studies [10, 15, 16]. The system vacuum was the same for all observations, and the system has no claw. Based on the strong relationship between MTVAC and AVGFLOW it seems possible to use average milk flow as a proxy for the vacuum level in the short milk tube during milking in an AMS. Increasing the system vacuum will increase the physical forces acting on the teat. Our findings indicates that cows with a low milk flow responded with poor teat-end condition even at a standard system vacuum level. Increasing system vacuum level is likely to increase the number of cows with this problem.

The PCA showed that MTVAC and COMPR were closely related. This indicates that COMPR and MTVAC contain similar information. Because COMPR accounts for both duration of the pulsation cycle, vacuum level in the pulsation tube and liner type, this variable might be better suited for comparisons between herds [12]. Since every milking was performed using the same liner and the same pulsation settings, and there was little variation in vacuum conditions in the pulsation tube between cows, COMPR was mainly influenced by the vacuum level in the short milk tube. The PCA also showed a negative relationship between the milk flow variables (AVGFLOW and PEAKFLOW) and COMPR, which is likely due to the strong association between the milk flow variables and vacuum level in the short milk tube.

In agreement with previous research on teat anatomy and milk flow rate in CMS [17], the PCA showed that there were no apparent association between teat dimensions and the milk-flow variables AVGFLOW and PEAKFLOW. Accordingly, no evident association was found between teat dimensions and the MTT-variables MTVAC and COMPR. The quarter milk flow rate has been shown to be a consequence of the canal anatomy, such as length and diameter, but sophisticated tools such as ultrasonography is required to acquire this kind of information [17].

The PCA also showed that teat dimensions were related to MPC vacuum; larger teat dimensions were associated with lower MPC vacuum. This finding is in agreement with results from a previous study performed in a CMS [19]; a high vacuum level in the MPC can be observed when the teat is too small relative to the diameter of the liner barrel, allowing the vacuum in the short milk tube to propagate to the MPC. A low vacuum level in the MPC is observed when the liner fits the teat, making a tight seal in the liner barrel. Low MPC vacuum levels may also be a result of air leakage due to the mouthpiece opening being too large relative to the base of the teat [19].

### Relationships between teat-end callosity and milk flow- and MTT-variables

The multivariable logistic regression models showed that MTVAC, COMPR, AVGFLOW, and PEAKFLOW were all associated with the outcome variables THICKNESS and ROUGHNESS. The negative relationship between the milk flow variables AVGFLOW and PEAKFLOW and the variables based on vacuum recordings; MTVAC and COMPR, as shown in the PCA (Fig. 1), is indicating that these four variables contain similar information.

Difference in BIC of two models < 2 is considered to be a weak evidence for superiority of the model with the lowest BIC, whereas values from 2 to 6 are considered positive evidence [26]. For both outcome variables, the models using AVGFLOW had the lowest BIC. However, the differences only provided weak evidence that the models using AVGFLOW was superior to the other. Nevertheless, this is a relevant finding because the milk flow is readily available in the herd management system and might be used instead of or in addition to vacuum measurements to indicate whether the milking procedure is involved as a cause for teat-end condition problems in a herd. If cows with poor teat-end condition also show low milk flow rates, it should be suspected that the milking settings (e.g. system vacuum) is not suited for this group of cows. In contrast, if poor teat-ends occur frequently across the whole range of milk flow rates, one might suspect that environmental and genetic factors are the dominating causes for the condition, or that the milking system has major defects affecting all cows. Further research is needed to test this hypothesis before it is implemented in herd health management protocols.

Because we used a cross sectional study design, it is relevant to ask whether the associations between teat-end callosity and milk flow rate could be interpreted in two directions; (1) milk flow affecting (vacuum levels and thereby) teat-end callosity, or (2) teat-end callosity affecting milk flow (and thereby vacuum levels). The length of the teat canal, measured by ultrasonography, has been shown to correlate with average and peak milk flow rate [17]. Our study showed that quarter average and peak milk flow rates were associated with vacuum levels in the short milk tube, which is in agreement with previous research from CMS [14]. Previous research have also shown that vacuum conditions at the teat-end during milking is involved in the development of teat-end callosity [10]. We therefore think it is plausible that the difference in development of teat-end callosity between cows primarily is a result of teat canal anatomy, manifested as differences in milk flow rate, rather than the opposite. However, we cannot rule out that a high degree of teat-end callosity may also act in concert with narrow teat canals, leading to further reduced milk flow rate in affected cows. Few authors, if any, have discussed the possible effects of teat-end callosity on milk flow rate.

Neijenhuis et al. [22] found less teat-end callosity in first parity cows than cows in third and later lactations. Although not statistically significant in our models, the OR estimates indicated a higher likelihood of having a thickened or roughened teat-end callosity ring in cows in third or later lactations compared to first lactation cows (Table 3). The cows in third or later lactations had been milked by CMS in two or more previous lactations. Although less likely, we cannot rule out that this have interfered with the degree of teat end callosity in the present investigation. Sterret et al. [29] investigated teat-end callosity in a group of Holstein cows before and after converting to quarter-based milking, and found a decrease in teat-end callosity approximately 1 month after installing the new system. This shows that teat-end callosity is a dynamic condition, and that previous milking machine settings might be of minor importance. Because our main focus was associations between teat-end callosity and milk flow, not prevalence of teat end callosity, we consider possible effects of earlier exposure to CMS to be of minor importance for our conclusions.

We used vacuum recordings to split the milking into two main phases; main milking and overmilking. Because this is an indirect method, it is not possible to determine exactly when the milk flow starts to decline and when it drops below the take-off limit, which might lead to some misclassification if compared to methods using data from milk meters. The duration of the main milking phase was used as the denominator in the calculation of MTVAC and MPCVAC. Furthermore, duration of the overmilking period was used both as an explanatory variable and as the denominator in the calculation of MPCOM. Thus, it is obvious that erroneous calculations of the transition between the main milking and overmilking periods would hamper the results of the present investigation. However, we have used accepted and standardized methods commonly implemented in herd health advisory services around the world [30]. It is also worth noting that both MTVAC and AVGFLOW were significantly related with teat-end callosity thickness and roughness, and that AVGFLOW was calculated by the AMS software independent of how we defined the transition between milking phases. This indicates that the definition of the main milking and overmilking phases cannot have had a major impact on our models. We also acknowledge that the varying time span between teat-end scoring and vacuum measurements and the non-standardized time from milking to teat measurement may have added variability to the results. It is reasonable to expect that less variability due to a more uniform sampling regime would strengthen rather than weaken the reported associations, which already by the present approach have shown to be quite significant.

No associations were found between THICKNESS and ROUGHNESS and overmilking time. This is likely due to short duration and little variation in the overmilking periods in AMS, as described in previous studies [4, 5]. In our data, 90% of the observations had an overmilking time between 16 and 48 s. Because the AMS settings included a low-vacuum period of 6 s and a delay from initiation of detachment to actual detachment of 4 s, there might be a slight overestimation of the duration of the overmilking time. However, this overestimation is similar for all milkings, and we do not expect this to bias our assessments of overmilking time and teat end condition.

A recent study revealed a relationship between MPC vacuum and congestion at the teat-ends [20]. Our outcome variables can be considered long-term changes in the teat tissue, whereas the congestion shown by Penry et al. [20] was observed by ultrasonography immediately after milking. Despite short overmilking periods, the variable MPCOM showed a significant association with THICKNESS in the univariable analysis (P < 0.05). However, none of the MPC variables showed significant associations with THICKNESS or ROUGHNESS in the multivariable models when DIM and parity were accounted for. Conclusively, further research is warranted to increase the knowledge on how overmilking in AMS can be evaluated by vacuum recordings.

The data were obtained from a single AMS herd in which all cows were milked with the same milking machine settings and the same liner, and we acknowledge that this may lower the external validity. However, because relationships between teat-end condition and milking machine performance has traditionally been studied in CMS [14–16, 31], our study in an AMS herd under field condition represents a step forward for the knowledge on associations between teat-end condition and milk flow in AMS herds. Due to the differences between CMS and AMS, e.g. milking cluster vs. individual attachment and detachment, our findings should be used with precaution in CMS.

A previous study have indicated that breeds differs concerning the development of teat-end hyperkeratosis [31]. Although we expect associations between milk flow variables in AMS and teat end condition to be similar also for other breeds than Norwegian Red, it is feasible to investigate these associations separately in other breeds.

## Conclusion

Quarter milk flow rate obtained from the AMS software may be used as a proxy for vacuum level in the short milk tube. Furthermore, quarter milk flow rate obtained from the AMS provided useful information for evaluating associations between the milking procedure and risk factors for impaired teat-end condition.
